# Management of acute ischemic stroke in the emergency department: optimizing the brain

**DOI:** 10.1186/s12245-024-00780-5

**Published:** 2025-01-07

**Authors:** Latha Ganti

**Affiliations:** 1 Orlando College of Osteopathic Medicine, Winter, FL 34787 USA; 2 Warren Alpert Medical School of Brown University, Providence, RI 02903 USA

**Keywords:** Acute ischemic stroke, Blood pressure management, Thrombolysis

## Abstract

Acute ischemic stroke is a devastating condition that afflicts more than 12 million people every year. Globally, stroke is the 2nd leading cause of death and 3rd leading cause of disability worldwide. While not all patients can avail themselves of existing acute therapies, all patients can benefit from brain optimization measures. This paper details the 12 steps in the management of acute ischemic stroke in the emergency department.

## Introduction

As of 2019, stroke claimed 3.29 million lives worldwide. That number is expected to increase further to 4.90 million by year 2030 [1]. Stroke is a leading cause of both death and disability worldwide, and the emergency department (ED) is often the initial point of care for these patients. The approach outlined in this manuscript provides a structured and comprehensive framework for managing AIS in the ED, highlighting brain optimization measures for patients who are not eligible for thrombolysis or thrombectomy. This focus on optimizing care for the vast majority of stroke patients—who do not qualify for acute interventions—addresses a significant gap in current stroke management protocols.

The only FDA-approved therapy for acute ischemic stroke (AIS) is tissue plasminogen activator (t-PA), based on the National Institutes of Neurologic Diseases Institute study [2]. Approved in 1996, t-PA is administered as alteplase at a dose of 0.9 mg/kg with 10% as a bolus and the remainder as an infusion. More recently, tenecteplase (at a dose of 0.4 mg/kg as a single bolus) is being used despite not having official FDA clearance for the treatment of AIS. This is due to the ease of administration and either equivalent or superior outcomes with regard to the rate of symptomatic hemorrhage, functional outcome at 90 days, and reperfusion grade after thrombectomy [3]. However, only 3–8.5% of potentially eligible patients receive thrombolytic therapy [4]. For patients with an arterial occlusion in the proximal middle cerebral artery or basilar artery, thrombectomy is another acute therapeutic intervention. A 2019 study performed at a comprehensive stroke center (CSC) found that only seven out of one hundred AIS patients were eligible for thrombectomy according to the 2018 guidelines by the American Heart Association (AHA), highlighting that a majority of AIS patients are thrombectomy-ineligible [5].

Considering the statistics above, approximately 90% of acute ischemic stroke patients do not receive any acute therapeutic options. Some of this gap may be corrected by improving patients’ knowledge about stroke [6], including providing language-specific education [7, 8]. Despite this relatively large proportion of patients ineligible or unable to obtain thrombolysis or thrombectomy, there are many things physicians can do to optimize the actively infarcting brain. This paper will review the 12 steps in the management of acute ischemic stroke after the patient arrives at the emergency department, with a focus on brain optimization measures. This paper does not discuss the many factors that influence *which* ED the patient is transported to.

## Review

**Fig. 1:**
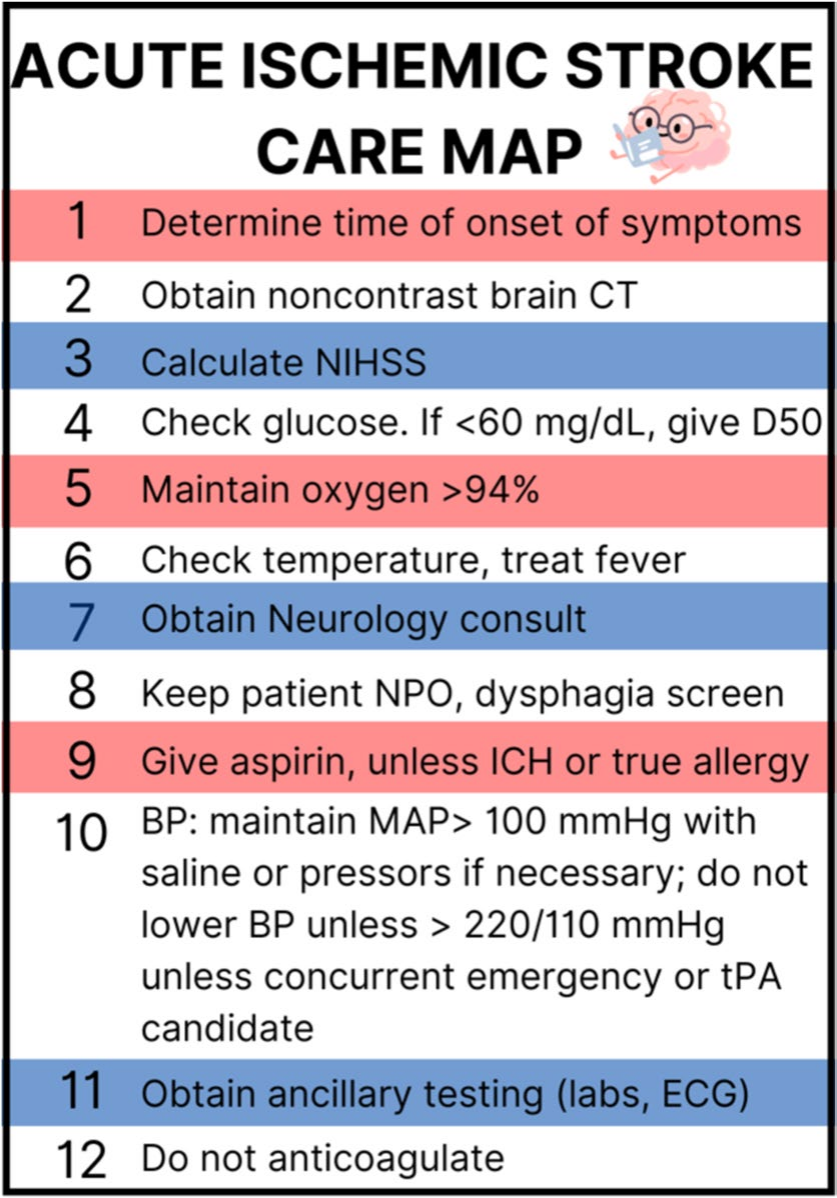
Acute stroke care map. Graphic designed by Latha Ganti on Canva.com. CT= computed tomography; NIHSS= National Institutes of Health Stroke Scale; D50= dextrose 50%; ICH= intracerebral hemorrhage; MAP= mean arterial pressure; ECG= electrocardiogram

The acute ischemic stroke care map is summarized in Fig. 1.

**Fig. 2:**
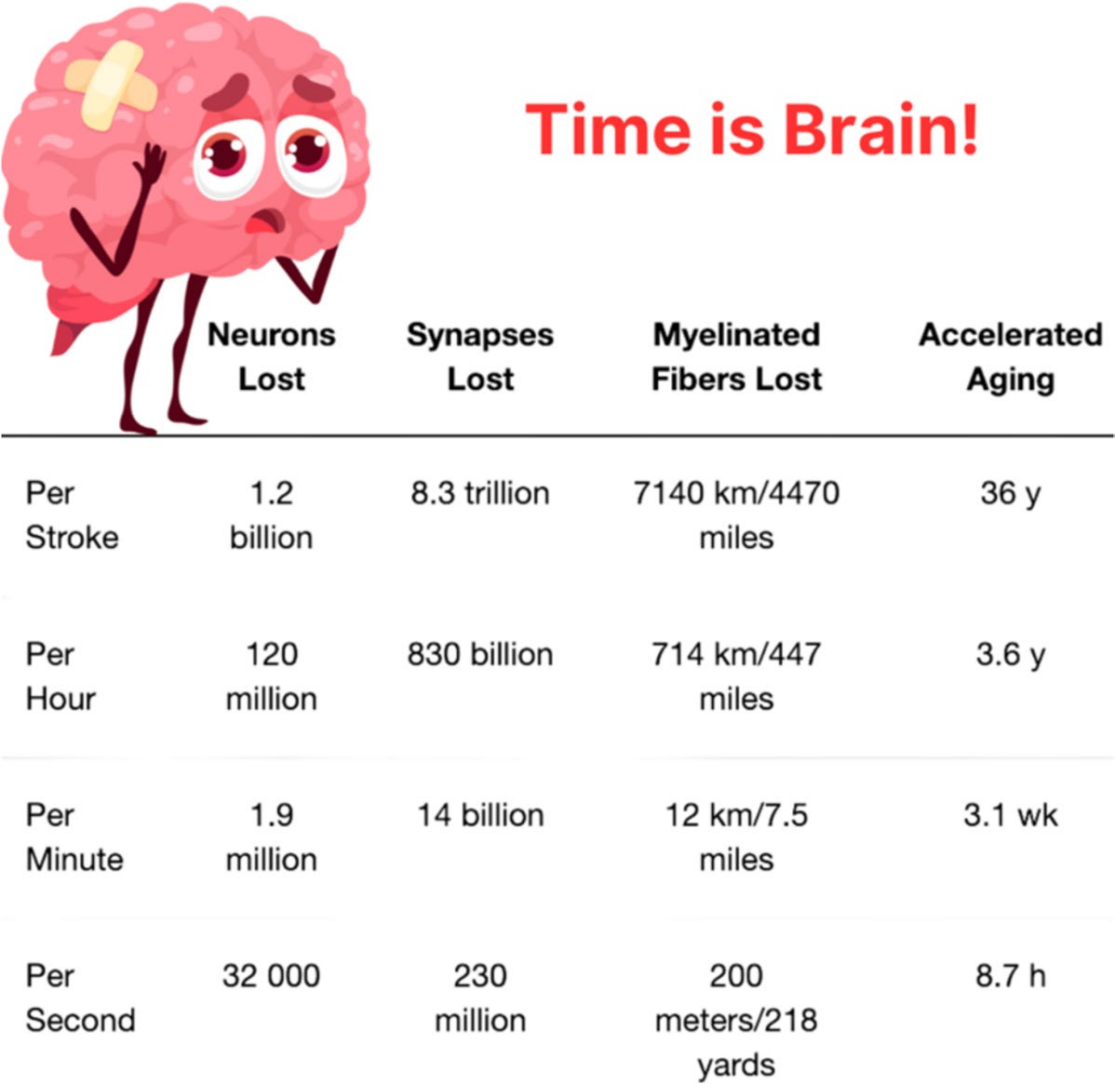
Time is Brain Quantified. Data from [9]. Graphic designed by Latha Ganti on Canva.com

Time is brain (Fig. 2) [9]. Therefore, the emergency department evaluation of the acute stroke patient must occur swiftly as well as accurately.

### Step 1: ascertain the time of onset of symptoms

The first step is to determine the time of onset of symptoms, as this effectively determines whether a patient is a tPA candidate or not. For patients who woke up with stroke symptoms, the time of onset is traced back to the last known well time, which would be when they went to bed. This is still the definition being used, even though there is good data to suggest that acute ischemic strokes, like myocardial infarctions, likely happen in the hour before awakening [10].

### Step 2: obtain non-contrast brain CT

The next step is to obtain a non-contrast brain CT. The purpose of this scan is to look for intracerebral hemorrhage (ICH), which is an absolute contraindication for thrombolysis. Only a non-contrast brain CT is needed to decide whether or not to administer tPA. CT angiography is used to look for occlusions amenable to thrombectomy, but advanced imaging should not delay tPA administration, as it is accepted that the earlier in their stroke course an eligible patient receives t-PA, the better their outcome [11].

### Step 3: Perform the National Institutes of Health Stroke Scale (NIHSS)

**Fig. 3:**
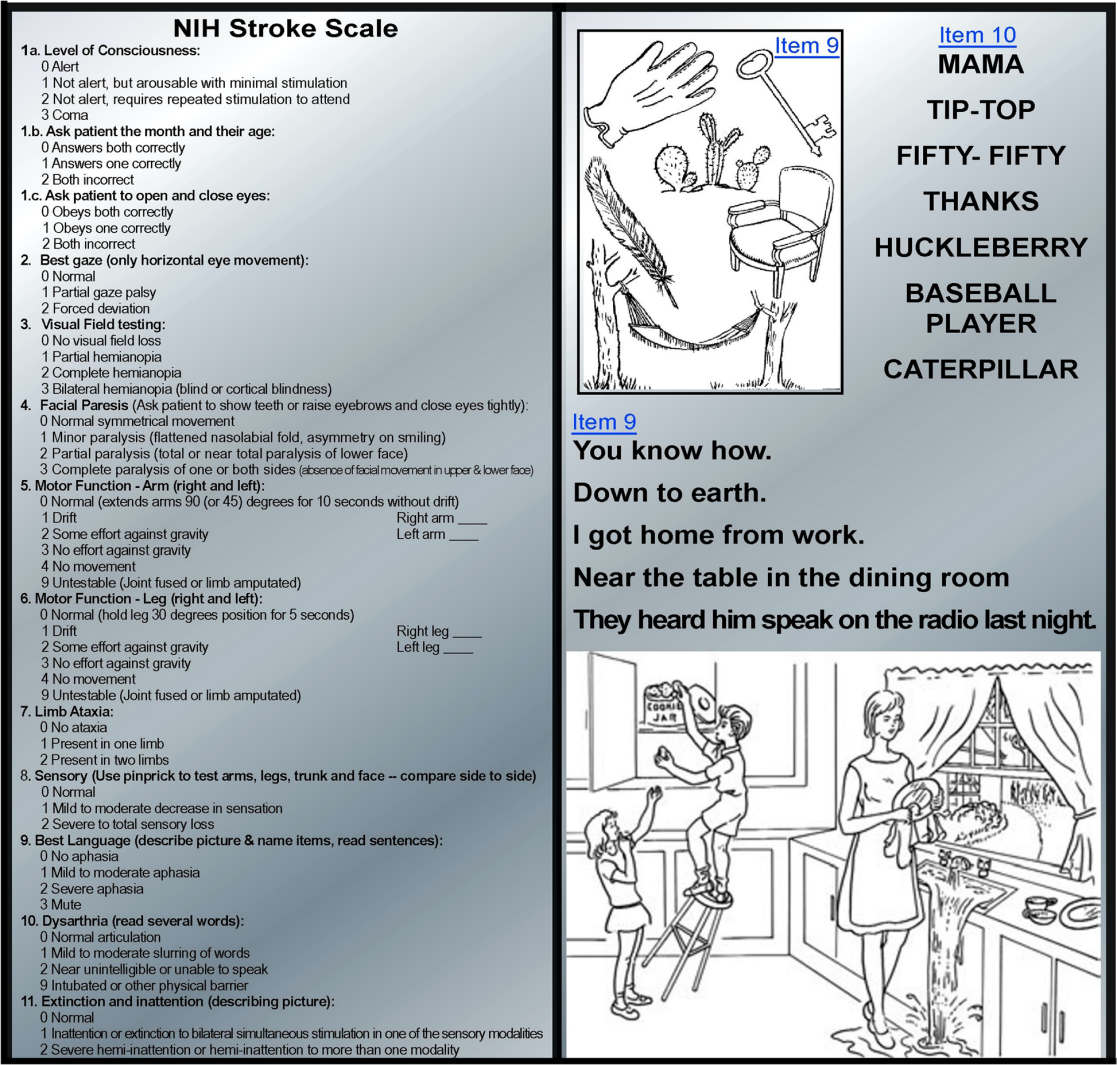
A summary of the National Institutes of Health Stroke Scale [10]

The clinical assessment (history, general examination, and neurological examination) remains the cornerstone of the evaluation. The use of a stroke rating scale, preferably the NIHSS, is recommended) [11]. The NIHSS is a 42-point scale, with higher numbers indicating worse neurologic deficits. This scale has been widely adopted in the United States, as part of hospital-wide stroke quality initiatives, and free training on the scale is available (Fig. 3).

Step 3a: If a patient is a tPA candidate, prepare thrombolytic for administration.

### Step 4: check rapid bedside glucose

#### Hypoglycemia

Glucose is the major cerebral energy substrate in the adult brain. It is critical for synaptic activity that relies on the complex metabolic interactions between neurons and astrocytes [12]. Hypoglycemia thus disturbs cerebral metabolism, and for this reason, the brain is considered especially vulnerable to hypoglycemia due to its high metabolic demand, reliance on glucose as a primary fuel, and minimal fuel stores [12]. Hypoglycemia can mimic ischemic stroke [13]; for this reason, a rapid glucose test is the only lab test that should be performed before administering intravenous tPA [11]. Recurrent hypoglycemia has also been shown to be detrimental to brain function [14].

#### Hyperglycemia

Hyperglycemia has been associated with adverse prognosis following AIS [15] and is considered a marker of a more severe stroke. A review of hyperglycemia in acute ischemic stroke [16] reports that experimental data suggest that elevated blood glucose may directly contribute to infarct expansion through several maladaptive metabolic pathways, and postulate that treatment with insulin may mitigate these adverse effects. However, a Cochrane review by the same team found no evidence that tight glycemic control with insulin resulted in improved outcomes. This review included 11 randomized controlled trials and 1583 patients and found no benefit in terms of functional outcome, death, or improvement in final neurological deficit but significantly increased the number of hypoglycemic episodes. Indeed, patients whose glucose levels were maintained within a tight range with intravenous insulin experienced a greater risk of symptomatic and asymptomatic hypoglycemia [17]. Interestingly, hyperglycemia at acute stroke presentation may often represent occult diabetes [18]. A Mayo Clinic study of 446 patients reports that patients with hyperglycemia exhibited significantly greater stroke severity (*P* = 0.002) and greater functional impairment (*P* = 0.004) than those with normoglycemia. Patients with hyperglycemia were 2.3 times more likely to be dead at 90 days compared to those with normal glucose (*P* < 0.001). Among the patients without a prior history of diabetes, patients with hyperglycemia were 3.4 times more likely to die within 90 days (*P* < 0.001) [18]. Persistent hyperglycemia (> 200 mg/dL) during the first 24 h is an independent predictor for ischemic stroke expansion and poor neurological outcome [11].

### Step 5: check room air oxygen saturation

The goal of oxygen therapy is to prevent hypoxia, which can potentially worsen the brain injury caused by ischemia. Partial airway obstruction, hypoventilation, aspiration pneumonia, and atelectasis are the most common causes of hypoxia in the AIS patient. Supplemental oxygen should be provided to maintain oxygen saturation > 94%. Nonhypoxic patients with acute ischemic stroke do not need supplemental oxygen therapy [11]. There is also no evidence that hyperbaric oxygen provides any benefit for the routine AIS patient. There is potentially a role for hyperbaric oxygen for a stroke secondary to air embolization [19].

### Step 6: check temperature

Pyrexia is a common phenomenon following AIS as well as intracerebral hemorrhage (ICH). It is associated with worse clinical outcomes, including longer length of stay (LOS), higher mortality, and disability [20].

A retrospective cohort study of 9366 patients with AIS found that peak temperature in the first 24 h < 37 °C and > 39 °C was associated with an increased risk of in-hospital death compared to normothermia [19].

The source of the fever should be ascertained. It could be secondary to concurrent infection such as pneumonia or urinary tract infection, or it may be a manifestation of cerebral disturbance, as is common with brainstem strokes. Regardless of etiology, it is important to treat fever. The best way to do this is with oral or rectal aspirin. The rectal route is preferred if a swallow screen is not cleared or there is any concern for dysphagia. Acetaminophen can also be used to treat fever and comes in both oral and rectal forms; however, aspirin has the added advantage of reducing the risk of future ischemic events. While targeted temperature management is popular for cardiac arrest, devices such as cooling helmets have not shown a clear benefit in AIS, based on a systematic review of 46 cooling helmet studies [21]. Controversial data exists regarding hypothermia and insufficient evidence exists to recommend hypothermia for the treatment of patients with AIS according to a Cochrane review of 426 patients [22].

### Step 7: neurology consultation (if available)

While the decision of whether or not to give tPA is well within the emergency physician’s domain, in a busy emergency department, having an additional colleague to help with the acute stroke patient can be extremely helpful. In a prospective cohort of 107 patients, an emergency department-based study reported that the lack of a dedicated stroke team was significantly associated with longer door-to-needle times for stroke management [23]. Consultation could occur in person or via telemedicine depending on the local hospital resources.

### Step 8: keep patients NPO (nothing per orem)

It is important to keep acute stroke patients NPO, because impairments of swallowing are associated with a high risk of aspiration pneumonia and increased risk of death. A review of 42 studies with 26,366 participants found that post-stroke dysphagia was associated with a higher pooled odds ratio (OR) for risk of pneumonia 4.08 (95% CI, 2.13–7.79) and mortality 4.07 (95% CI, 2.17–7.63) [24]. Patients with infarctions of the brain stem, multiple strokes, major hemispheric lesions, or depressed consciousness are at the greatest risk for aspiration [25]. Risk factors for aspiration include an abnormal gag reflex, impaired voluntary cough, dysphonia, incomplete oral-labial closure, more severe strokes, and cranial nerve palsies. It is important to note that a preserved gag reflex may not indicate safety with swallowing.

### Step 9: Antiplatelet agents

A Cochrane review covering 11 studies involving 42,226 participants reported that treatment with aspirin 160–325 mg resulted in decreased odds of death or dependency at six-month follow-up. The number needed to treat is 79, so for every 1000 people treated with aspirin, 13 people would avoid death or dependency [26]. For patients who have an ischemic stroke while already on aspirin therapy, an alternate antiplatelet agent such as clopidogrel is given. Studies have also investigated the use of dual antiplatelet therapy (DAPT). A Cochrane review encompassing 15 studies and 17,091 patients found that DAPT was more effective in reducing stroke recurrence but increased the risk of hemorrhage compared to a single antiplatelet agent. However, the systematic review concluded that the benefit in the reduction of stroke recurrence seems to outweigh the harm of dual antiplatelet agents initiated in the acute setting and continued for one month [27]. Based on these data, the AHA recommends that in patients presenting with a high risk minor stroke or transient ischemic attack, treatment for 21 days with dual antiplatelet therapy (aspirin + clopidogrel) initiated within 24 h can be beneficial for early secondary stroke prevention for a period of up to 90 days from symptom onset [11].

### Step 10: blood pressure management

The normal cerebral blood flow (CBF) in the human brain is approximately 50–60 mL per 100 g of tissue per minute [28]. When cerebral blood flow drops to 50%, synaptic transmission stops, and cerebral energy use decreases by 50%. This represents the penumbra area of an infarct. At this point, autoregulation fails and the ischemic brain becomes dependent on systemic circulation for perfusion [29]. Autoregulation is the brain’s ability to maintain its cerebral perfusion pressure irrespective of what is happening to the systemic blood pressure. Once autoregulation fails, cerebral perfusion pressure (CPP) is directly dependent on systemic blood pressure [30]. Therefore, decreasing systolic blood pressure in acute ischemic stroke can be detrimental to the ischemic brain, extending the area of infarct. This is why one should not acutely decrease blood pressure in acute ischemic stroke. Emergency administration of an antihypertensive agent should be withheld in ischemic stroke unless the systolic blood pressure is greater than 220 mm Hg or the diastolic blood pressure is greater than 120 mm Hg. Additional indications for acute antihypertensive administration would include if there is a concurrent hypertensive emergency (such as acute aortic dissection) or if the patient is a tPA candidate [11]. If the patient is a t-PA candidate, then the blood pressure should be lowered to 185/110 mmHg. When a blood pressure-lowering agent is given, it should be lowered no more than 15% in the acute period. Several antihypertensive agents are available [Table 1], but none of them have been compared in randomized controlled trials. Rather, they are consensus-based guidelines. Agents that are not recommended for blood pressure treatment in acute ischemic stroke are hydralazine and nitroglycerin, due to their vasodilatory properties that can result in an extension of the area of infarct. Hydralazine raises intracranial pressure, which, together with its effect upon systemic blood pressure, reduces cerebral perfusion pressure [31]. It is important to note that in the majority of patients, a decline in blood pressure occurs within the 1st few hours after stroke without any specific medical treatment.

**Table 1:** Antihypertensive drug options for treating hypertension in acute ischemic stroke (> 220/110 mmHg in non-tPA candidates and > 185/105 mmHg in t-PA candidates; caveats for concurrent select hypertensive emergencies)

Drug	Dose	Onset of action	Elimination half life	Duration of action	Contra-indications	Caution
Labetalol	10–20 mg IV over 1–2 min, may repeat 1 time	2–5 min	5.5 h	4 h	Bradycardia; 2nd or third-degree heart block, severe asthma	
Clevidipine	1–2 mg/h IV, titrate by doubling the dose every 2–5 min until desired BP reached; maximum 21 mg/h	2–4 min	1 min	5–15 min		Lipid emulsification can result in hypertriglyceridemia
Nicardipine	5 mg/h IV, titrate up by 2.5 mg/h every 5–15 min, maximum 15 mg/h; when desired BP reached, adjust to maintain proper BP limits	5–10 min	40–60 min	15–90 min	Severe aortic stenosis	Cumulative doses can result in cyanide toxicity
Enalaprilat	1.25 mg/dose given over 5 min every 6 h	< 15 min	35 h	6 h	history of angioedema related to an ACE inhibitor; bilateral renal artery stenosis	Not easily titratable, long half life

**Fig. 4:**
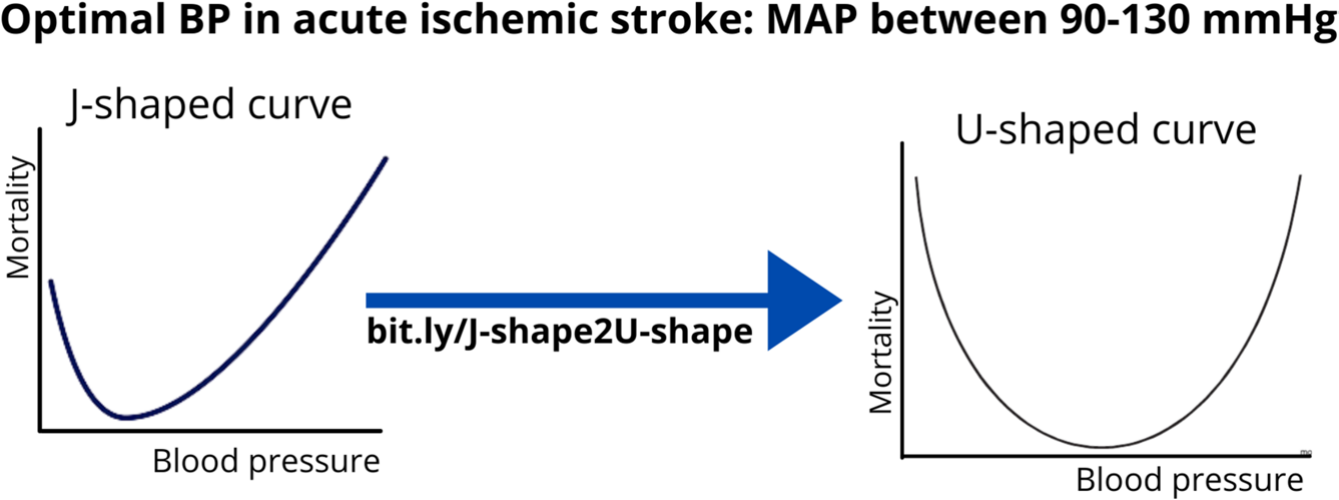
The “sweet spot” for blood pressure in acute ischemic stroke follows a U-shaped curve [32]

In addition to elevated blood pressure, the lower end of blood pressure is also equally important. A Mayo Clinic study of 357 consecutive ED patients was one of the first to demonstrate the detrimental impact of low pressure on acute stroke outcomes. The study reported that patients with low BP (dBP < 70, sBP < 155, or MAP < 100 mm Hg) were significantly more likely to die within 90 days than those with BP in the normotensive or mildly hypertensive range (dBP 70 to 105, sBP 155 to 220, MAP 100 to 140 mm Hg). These associations were significant even after adjusting for age, sex, and NIHSS score [32]. This led to the paradigm shift of thinking of the blood pressure and stroke outcome as a U-shaped curve, rather than a J-shaped curve (Fig. 4). This study was followed by several other similar studies leading to the updated American Stroke Association (ASA) recommendation supporting the treatment of low blood pressure with intravenous fluids and or pressors as needed to maintain systemic perfusion levels necessary to support organ function [11].

### Step 11: Obtain ancillary testing

Ancillary studies can decipher systemic conditions that may mimic or cause stroke or identify systemic conditions that may influence therapeutic options. Routine labs in the evaluation of acute ischemic stroke include a complete blood count (CBC), electrolyte panel, liver function tests (LFTs), coagulation studies, and sometimes troponin level measurement. Many patients receive their primary diagnosis of hypertension, diabetes, hypothyroidism, or heart disease at the time of presentation for acute stroke. Because the medications utilized to treat these conditions are cleared either through the kidney or the liver, having a chemistry panel and LFTs are useful to provide a baseline. Determination of the platelet count and, in patients taking warfarin or with liver dysfunction, the prothrombin time (PT) and international normalized ratio (INR) is important. A CBC can reveal leukocytosis or anemia, both of which can exacerbate symptoms or provide clues to other underlying conditions. An electrocardiogram is also routinely obtained in an acute stroke evaluation.

### Step 12: do not anticoagulate

Until about 20 years ago, acute ischemic strokes would receive prompt anticoagulation. Since then, a Cochrane review including 28 trials involving 24,025 participants [33] has informed us that early administration of anticoagulants does not lower the risk of early recurrent stroke, even among patients with cardioembolic stroke. Anticoagulants do not lessen the risk of neurological worsening. There is also no adequate data to demonstrate the efficacy of anticoagulants in potentially high-risk groups such as patients with intracardiac or intraarterial thrombi, making no early anticoagulation a level I recommendation by the American Stroke Association (ASA) [11]. Current data do not support the routine use of any of the currently available anticoagulants for acute ischemic stroke.

#### Thrombolytics

Patient who present within 4.5 h of symptom onset should be considered for thrombolytic therapy. Indications include age of 18 years or older, being within the drug time window, and having signs and symptoms compatible with a stroke. Several contraindications to thrombolysis exist [Table 2].

**Table 2:** Contraindications to t-PA for acute ischemic stroke

Evidence of intracerebral hemorrhage (ICH) on non-contrast brain CT scan or any history of ICH in the past
Ischemic stroke within the last 3 months
Severe head trauma within 3 months
Signs and symptoms consistent with subarachnoid hemorrhage
Gastrointestinal (GI) malignancy or GI bleed within 21 days
Coagulopathy: platelet count < 100,000/mm3, INR > 1.7, apTT > 40 s, PT > 15 s
Dose of low molecular weight heparin taken in last 24 h
Dose of direct thrombin inhibitor or factor Xa inhibitor taken in last 48 h
Presence of infective endocarditis, intracranial neoplasm, or aortic arch dissection

Recommendations summarized from [10]. *CT* Computed tomography, *INR* International normalized ratio, *aPTT* activated partial thromboplastin time, *PT* Prothrombin time

**Fig. 5:**
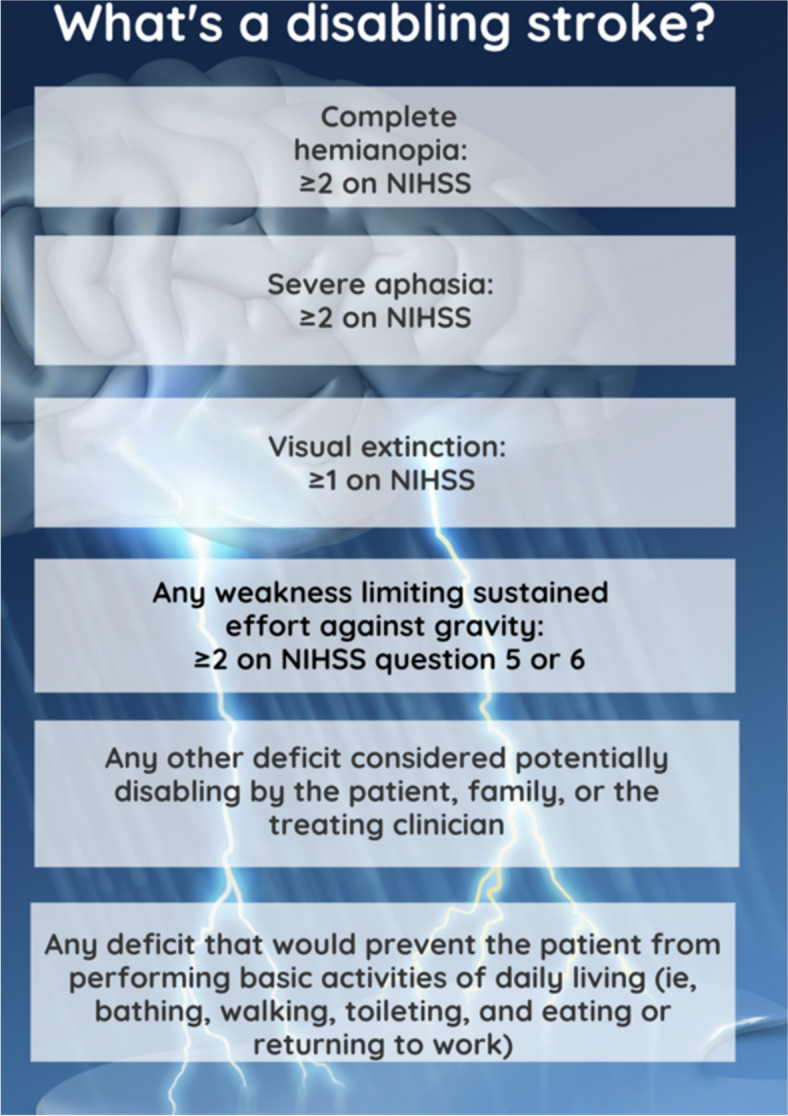
Elements of a non-disabling stroke. Infographic designed by Latha Ganti on Canva.com based on data from [34]

Over the last few guideline iterations, the number of contraindications for t-PA has decreased, leading to more widespread use. For the first time, however, this latest guideline [11] *adds* acontraindication – the presence of a mild *non-disabling* stroke; administering t-PA to these patients is not recommended as it may actually cause harm. This recommendation is informed by the PRISMS study, a double-blind, controlled, randomized, multicenter trial of 313 patients with NIHSS 0–5 and non-disabling stroke presentations with a baseline modified Rankin score of 0–1. This study showed no clear benefit with tPA over aspirin for non-disabling strokes and an increased risk for brain hemorrhage with tPA [34]. While it is sometimes difficult to define what is non-disabling, there is agreement on what constitutes a disabling stroke (Fig. 5).

This was followed by a study of an Austrian Stroke Unit prospective registry (ASUR) comprising 39 Austrian stroke units, *N* = 140,000, that dichotomized patients into those with mild strokes NIHSS 0–1 and NIHSS 2–5 [35]. This study reports that early neurological deterioration occurred in 10% of the intravenous thrombolysis (IVT) arm vs. no-IVT arm, patients undergoing IVT experienced more significant shifts in mRS and had increased mortality after 8 months and were less likely to have a NIHSS of 0 at discharge and mRS of 0 at 3 months.

**Fig. 6:**
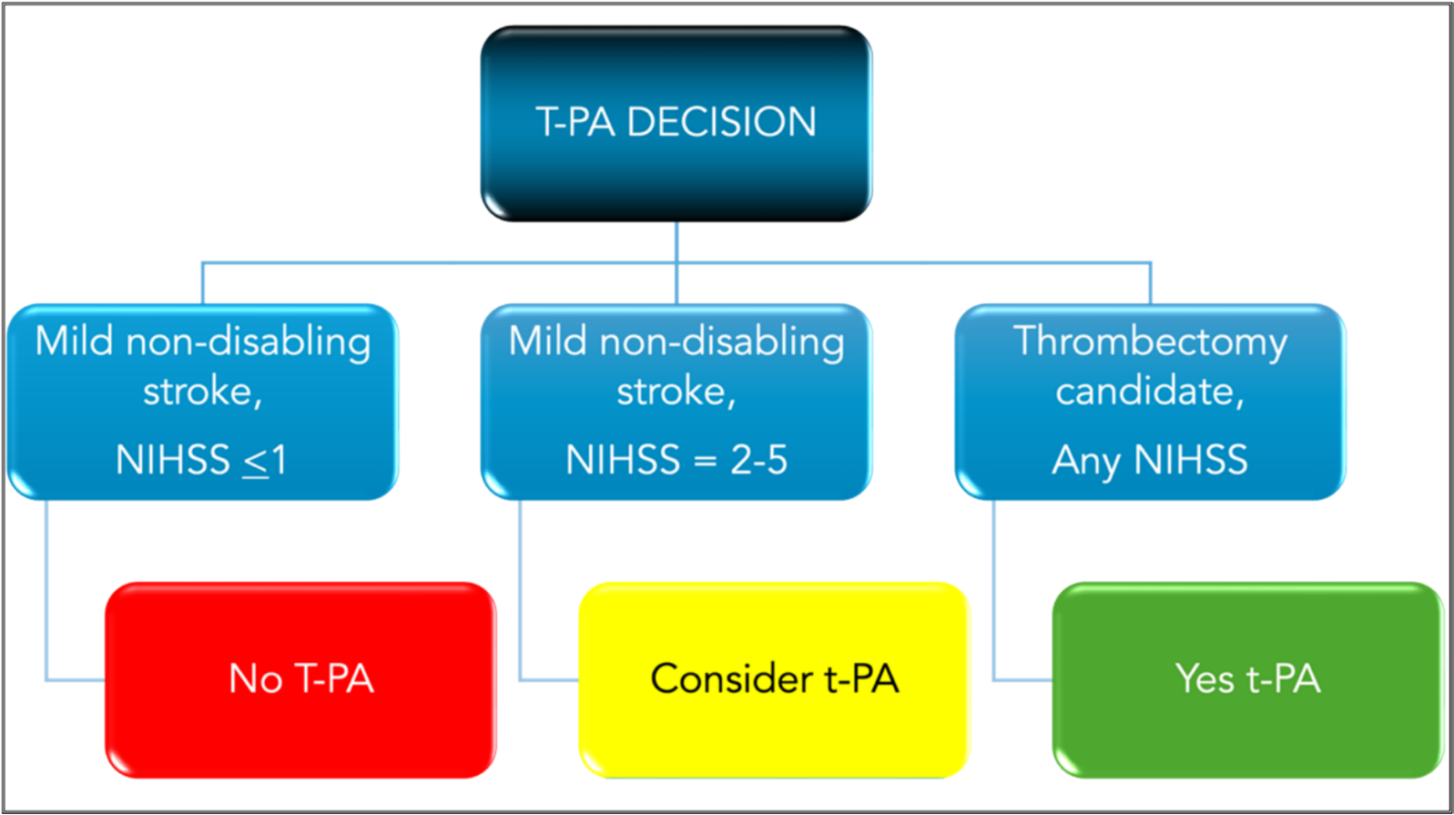
algorithm for treatment of mild acute ischemic strokes with NIHSS < 6

The MaRISS study [36] also examined mild strokes in 1736 patients with NIHSS 0–5 using registry-based data from over 100 hospitals in the Get with the Guidelines^®^ database. After adjusting for age, sex, race/ethnicity, and baseline NIHSS, investigators did not identify an effect of alteplase on the primary outcome (mRS 0–1 at 90 days) but did find an association with Stroke Impact Scale-16 in the restricted sample of baseline NIHSS score 3–5. Figure 6 summarizes the t-PA treatment decision for mild strokes.

**Fig. 7:**
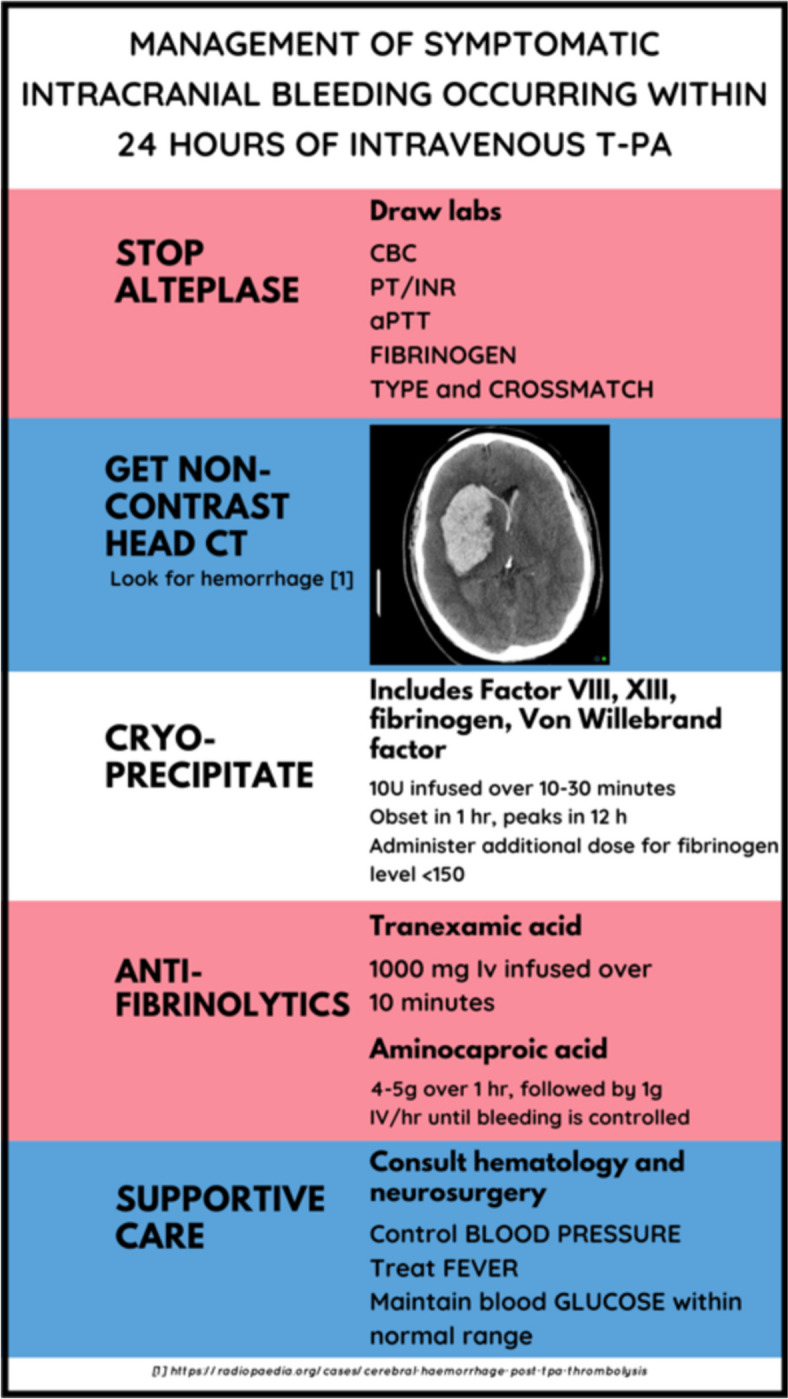
Management of symptomatic intracranial hemorrhage in the first 24 h after alteplase administration. Infographic designed by Latha Ganti on Canva.com based on data from [11]

One known and feared complication of thrombolysis in AIS is symptomatic intracranial hemorrhage [37]. The typical incidence quoted is ~ 6% [2], but this risk is higher for those with certain co-morbidities [38]. A 2023 systematic review of 105 studies concluded that there is moderate level evidence for the following being associated with a greater risk of ICH after thrombolysis for AIS: higher NIHSS, older age, diabetes, hyperglycemia, difficult-to-control hypertension, early ischemic changes on initial CT, larger areas of infarct, atrial fibrillation, antiplatelet therapy, or anticoagulant therapy. This review also found the same moderate level of evidence for leukoaraiosis, proteinuria, and elevated levels of fibrinogen, creatinine, and homocysteine, which are traditionally not included in the standard risk assessment [39]. When symptomatic ICH following thrombolysis does occur, a non-contrast brain CT is obtained to ascertain the extent of bleeding. Cryoprecipitate and antifibrinolytic therapy are given to control hemorrhage. Brain optimization measures discussed above are instituted, and specialty consultations are obtained as appropriate (Fig. 7).

Rarely, t-PA administration can result in an allergic reaction, usually manifested as angioedema. In this case, the t-PA (alteplase or tenecteplase) is immediately discontinued, if still infusing. This is followed up by standard ED treatment of histamine-mediated allergic reactions including 125 mg methylprednisolone IV, 50 mg diphenhydramine IV, and sometimes an intravenous H_2_ blocker. Angioedema can also result from a bradykinin-mediated mechanism, which is treated with a C1-esterase inhibitor, ecallanatide, or icatibant, as available. Fresh frozen plasma may be given if no bradykinin-mediated angioedema medications are available [40].

## Conclusion

An acute ischemic stroke is the ultimate medical emergency with more than a million neurons lost every minute. Thrombolysis and thrombectomy are two acute interventions with demonstrated efficacy. However, these are only options for a select minority of emergency department AIS patients – those who are at the right place at the right time. Patients need to present within a few hours of symptom onset and/or have a clot in a location amenable to thrombectomy. Nonetheless, brain optimization measures, including optimizing blood pressure, glucose, oxygen, and temperature and being mindful of ancillary studies are part of the acute stroke care map that can benefit *all* stroke patients.

## Data Availability

No datasets were generated or analysed during the current study.
